# Optimization of the Sintering Densification, Microstructure, Mechanical Properties, and Oxidation Resistance of Tib_2_–Tic–Sic Composite Ceramics via a Two-Step Method

**DOI:** 10.3390/ma18143297

**Published:** 2025-07-13

**Authors:** Fei Han, Wenzhou Sun, Youjun Lu, Junqing Ma, Shidiao Xu

**Affiliations:** 1 College of Materials Science and Engineering, North Minzu University, Yinchuan 750021, China; hanfei@nun.edu.cn (F.H.); chemlab2025@163.com (J.M.); xsd980919@163.com (S.X.); 2 Key Laboratory of Polymer Materials and Manufacturing Technology, North Minzu University, Yinchuan 750021, China; 3 National and Local Joint Engineering Research Center of Advanced Carbon-Based Ceramics Preparation Technology, Yinchuan 750021, China

**Keywords:** TiB_2_–TiC–SiC composite ceramics, boron/carbon thermal reaction, mechanical properties, oxidation resistance

## Abstract

In this investigation, TiB_2_–TiC composite powders, synthesized via the boron/carbon thermal reduction process, were employed as precursor materials. SiC, serving as the tertiary constituent, was incorporated to fabricate TiB_2_–TiC–SiC composite ceramics utilizing spark plasma sintering technology. The present study initially elucidates the densification mechanisms and investigates the influence of sintering temperature on the densification behavior, microstructural evolution, and mechanical properties of the resultant ceramics. The experimental findings reveal that the sintering process of TiB_2_–TiC–SiC ceramics exhibits characteristics consistent with solid-phase sintering. As the sintering temperature escalates, both the relative density and mechanical properties of the ceramics initially improve, reaching a maximum at an optimal sintering temperature of 1900 °C, before subsequently declining. Microstructural examinations conducted at this optimal temperature indicate a homogeneous distribution of the two primary phases, with no evidence of excessive grain growth. Furthermore, this research explores the effects of SiC addition on the mechanical performance and oxidation resistance of TiB_2_–TiC–SiC composite ceramics. The results demonstrate that the incorporation of SiC effectively suppresses grain growth and promotes the formation of rod-like TiB_2_ microstructures, thereby enhancing the mechanical attributes of the ceramics. Additionally, the addition of SiC significantly improves the oxidation resistance of the composite ceramics compared to their TiB_2_–TiC binary counterparts

## 1. Introduction

The TiB_2_–TiC composite ceramics composed of these two components not only amalgamate their individual strengths but also demonstrate enhanced physicochemical properties. Consequently, they are widely utilized in various high-temperature applications, including high-temperature crucibles, aerospace high-temperature components, and protective materials for nuclear reactors [1,2,3,4,5,6]. Nevertheless, due to their strong covalent bond coordination and low self-diffusion coefficients, it is difficult to achieve densification at lower temperatures [7,8,9]. Furthermore, at high temperatures, boride and carbide grains undergo significant growth, leading to a substantial decline in the material’s antioxidant performance above 1000 °C. These limitations hinder their practical engineering applications [10,11,12]. Therefore, enhancing the sinterability, fracture toughness, and oxidation resistance of TiB_2_–TiC composite ceramics remain critical technical challenges that must be addressed.

In light of the aforementioned challenges, incorporating additional components into the TiB_2_–TiC matrix to form multiphase composites represents a widely adopted and effective strategy. Within the realm of structural ceramics, SiC is frequently introduced as a reinforcing phase in TiC or TiB_2_-based systems [13,14,15]. For instance, Asl et al. [16] integrated SiC whiskers (SiCw) into a TiC matrix, achieving a notably higher relative density in samples containing >10 vol.% SiCw compared to monolithic TiC ceramics. Similarly, Oguntuyi et al. [17] added SiC to a TiB_2_ matrix, observing improvements in microstructural refinement and mechanical properties with increasing SiC content, culminating in an optimal SiC inclusion level of 20 vol.%. Pang et al. [18] fabricated TiB_2_–SiC composites via pressureless sintering, using TiB_2_ as the base matrix and SiC as the primary reinforcing phase. Their findings demonstrated that elevated SiC content progressively reduced grain size; minimized open porosity; and enhanced flexural strength, fracture toughness, and Vickers hardness. Conversely, TiB_2_ is also commonly employed as a toughening phase in SiC-based ceramics. Fattahi et al. [19] incorporated TiB_2_ into a TiC–SiCw composite, achieving peak flexural strength (511 MPa) and hardness (27.67 GPa) at a 30 vol.% TiB_2_ concentration. Huang et al. [20] investigated the effects of TiB_2_ addition on the microstructure and mechanical properties of SiC-based composites, revealing that TiB_2_ induced a transition of the grain boundary phase from crystalline to amorphous. This transformation strengthened intergranular bonding and improved the material’s mechanical performance.

The integration of SiC as a tertiary constituent in TiB_2_–TiC–SiC ternary composite ceramics offers a promising solution to existing challenges. Monolithic TiB_2_ ceramics exhibit superior fracture toughness and flexural strength compared to TiC or SiC, while SiC and TiC demonstrate superior high-temperature oxidation resistance relative to TiB_2_. Furthermore, SiC possesses exceptional wear resistance and hardness when juxtaposed with TiC and TiB_2_. By synergistically combining the advantageous properties of these three components, TiB_2_–TiC–SiC composite ceramics enable the development of materials characterized by enhanced high-temperature strength, improved oxidation resistance, and notable fracture toughness [21,22,23]. Currently, extensive research has focused on the fabrication techniques for TiB_2_–TiC–SiC ternary composite ceramics. For instance, Venkatesh et al. [24] utilized ultrafine SiC, TiC, and TiB_2_ powders to synthesize TiB_2_–TiC–SiC composite ceramics via spark plasma sintering (SPS) at 1800 °C. The resulting materials exhibited commendable mechanical properties; however, the elevated sintering temperature and the high cost of raw materials pose significant practical limitations. Alternatively, Sun et al. [25] employed Si powder, metal Ti powder, and B_4_C as precursors to prepare TiB_2_–TiC–SiC composite ceramics through in situ reactive sintering under hot isostatic pressing (HIP) conditions. Nevertheless, this approach not only requires sophisticated equipment but also yields ceramics with a relative density of only 92.67% due to residual oxide impurities. In summary, no existing fabrication method fully satisfies the performance requirements for practical applications, underscoring the necessity for further exploration of innovative preparation techniques and processes.

This study introduces a novel two-step fabrication method for TiB_2_–TiC–SiC ternary composite ceramics. Initially, high-purity, ultrafine TiB_2_–TiC composite powders with a tunable two-phase composition are synthesized via the boron/carbothermal reduction process. Subsequently, a precise quantity of SiC powder is introduced, and spark plasma sintering (SPS) is employed to consolidate the TiB_2_–TiC–SiC ternary composite ceramics. Compared to existing approaches for preparing TiB_2_–TiC–SiC composite powders, our method offers two distinct advantages. First, the reaction precursors (B_4_C, TiO_2_, and carbon powder) are cost-effective, excluding silicon carbide. Second, this work leverages our group’s prior research, wherein we successfully synthesized TiB_2_–TiC composite powders with oxygen impurity levels below 1 wt.% and grain sizes controlled within 200 nm using the boron/carbothermal reduction technique. The low oxygen content mitigates the formation of gaseous byproducts during sintering densification, which could otherwise impede mass transport during consolidation. Furthermore, the use of micro/nanoscale powders significantly accelerates mass transfer at grain boundaries, facilitating the achievement of high-density ceramics in subsequent processing steps. By optimizing both the fabrication process and material composition, the resulting TiB_2_–TiC–SiC composite ceramics exhibit superior performance, thereby addressing key challenges in the field.

## 2. Materials and Methods

**Figure 1:**
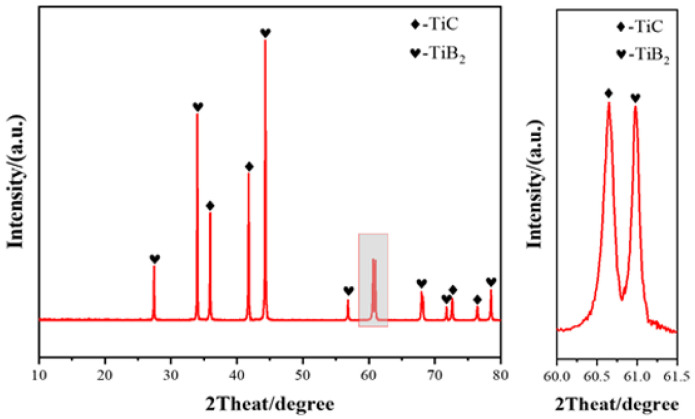
XRD diffraction pattern of TiB_2_–TiC powder.

**Figure 2:**
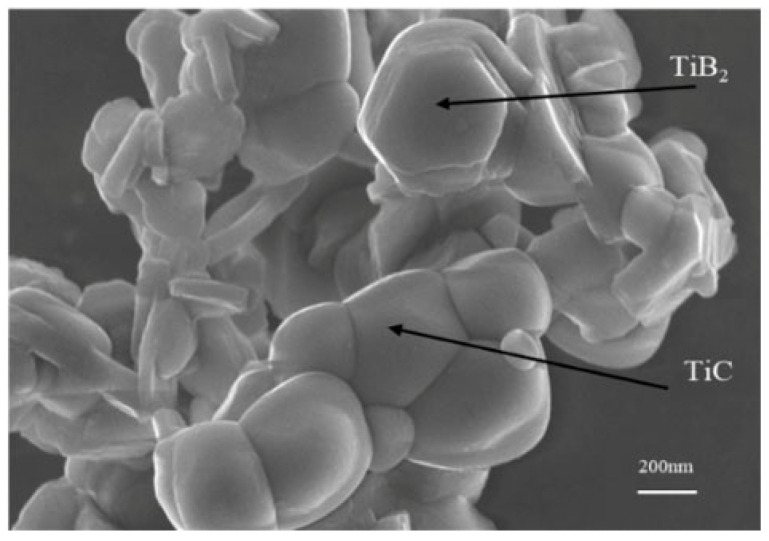
SEM image of TiB_2_–TiC powder.

In this study, TiB_2_–TiC composite powder synthesized via the boron/carbon thermal reaction method was used as the precursor powder; the reaction temperature was set at 1400 °C; the atmosphere was maintained with Ar gas; and the primary raw materials utilized were TiO_2_ (50 nm, 99.9% purity, sourced from Shanghai Gangtian Nano Materials Co., Ltd., Shanghai, China), B_4_C (50 nm, 99.9% purity, also from Shanghai Gangtian Nano Materials Co., Ltd., Shanghai, China), and carbon black (50 nm, 99.9% purity, likewise provided by Shanghai Gangtian Nano Materials Co., Ltd.) powders. A certain proportion of SiC powder (50 nm, 98.99% purity, purchased from Shanghai Gangtian Nano Co., Ltd., Shanghai, China) was added to prepare TiB_2_–TiC binary and TiB_2_–TiC–SiC ternary composite ceramics. The XRD diffraction pattern and SEM image of the self-made composite powder are shown in Figure 1 and Figure 2, respectively.

The phase composition of the sample was determined to be cubic TiC and hexagonal TiB_2_, corresponding to the PDF standard cards 35-0741 and 32-1383 in the ICSD database, respectively. The grain size of the TiB_2_ and TiC phases is approximately 100–200 nm. A phase quantitative analysis was performed using the GSAS-II software (2.0 Version, Advanced Photon Source, Argonne, IL, USA) based on the Rietveld refinement principle. The mass percentages of TiB_2_ and TiC in the composite powder were 64.1% and 35.9%, respectively.

In the present study, composite ceramics were sintered using an SPS sintering furnace (Shanghai Chenhua Technology Co., Ltd., Shanghai, China). The sintering process, detailed in Table 1, was carefully designed. Initially, the experiment investigated the impact of sintering temperature on the densification process and material properties, ultimately determining the optimal sintering conditions. Throughout this experimental, the SiC addition was maintained at a constant 15 wt%. The heating rates were adjusted at different stages: 80 °C/min from room temperature to 1200 °C, 45 °C/min between 1200 °C and 1650 °C, and 25 °C/min above 1650 °C. Once the desired temperature was reached, a constant pressure of 40 MPa was maintained for 10 min. The ceramic samples were initially cut into standard test specimens using wire electrical discharge machining (DK7740, Suzhou Sanguang Technology Co., Ltd. Suzhou, China). Subsequently, an automatic grinding machine was employed for rough grinding and fine grinding of the sample surfaces, followed by surface polishing with a precision polishing machine. After determining the optimal sintering temperature, this article further explores the influence of silicon carbide on the microstructure, mechanical properties, and oxidation resistance of composite ceramics. The raw material formulations are listed in Table 2.

The phase composition of the samples was analyzed with an X-ray diffractometer (XRD–6000, Shimadzu, Kyoto, Japan), utilizing a Cu target and a scanning rate of 1°/min. The microstructure of the polished ceramic surfaces and fracture morphologies were observed using scanning electron microscopy (SEM; SIGMA 500, ZEISS, Oberkochen, Germany). Elemental distribution within the samples was characterized by EDS mapping. The bulk density of the ceramics was ascertained by applying Archimedes’ principle, enabling the calculation of relative density. Hardness measurements of the multiphase ceramics were conducted using a Vickers hardness tester (HV–30BZ, Taile Co., Ltd., Changzhou, China) with a test load of 98N and a 10-s dwell time. Fracture toughness was determined using Equation (1), where P denotes the applied load, and C_1_ and C_2_ represent the diagonal crack lengths.

(1) $$K_{IC}=P{\left[\pi \left(\frac{C_{1}+C_{2}}{4}\right)\right]}^{-(\frac{3}{2})}{\left(tan\beta \right)}^{-1}$$

The three-point bending strength of ceramics was evaluated using a universal testing machine. Samples of the multiphase ceramics were cut into strips with dimensions of 3 mm × 4 mm × 20 mm by wire electrical discharge machining (WEDM), polished, and chamfered using sandpaper. The test was conducted with a span of 16 mm. The formula for calculating the bending strength is as follows:

(2) $$\sigma =\frac{3FL}{2bh^{2}}$$

In this formula, F denotes the peak load at which the ceramic specimen experiences bending failure, L represents the span length, while b and h correspond to the width and height of the sample, respectively. The static oxidation resistance method was utilized to assess the oxidation resistance of the samples. The samples were precisely cut into 5 × 5 × 5 mm cubes, polished to a smooth finish, thoroughly cleaned, and accurately weighed. Following this, the samples underwent oxidation in a muffle furnace, exposed to temperatures spanning from 800 °C to 1500 °C. Each temperature point was maintained for a duration of 8 h. Upon the samples’ cooling, they were re-weighed, enabling the plotting of an oxidation weight gain curve.

## 3. Results and Discussion

### 3.1. Sintering Densification

**Figure 3:**
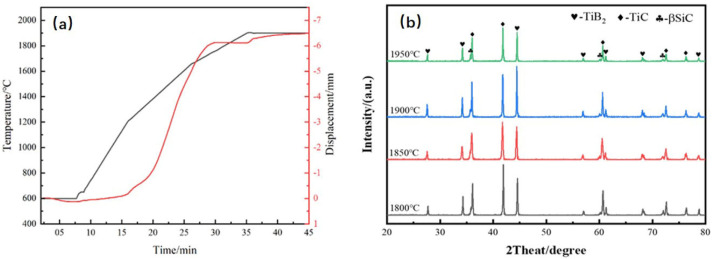
TiB_2_–TiC–SiC composite ceramic: (**a**) sintering densification curve; (**b**) XRD patterns.

The progress of sintering densification can be inferred based on the changes in the displacement of the indenter. The temperature and indenter displacement curves during sintering are shown in Figure 3.

As illustrated in Figure 3a, the sintering process of composite ceramics can be delineated into three distinct stages: Stage I occurs below 600 °C. During this initial phase, the upward displacement of the indenter is primarily attributed to the thermal expansion of both the powder compact and the graphite die upon heating. This thermal expansion results in mechanical displacement of the indenter without significant densification. Stage II spans from 600 °C to 1800 °C. This stage is critical for ceramic densification, marked by a substantial downward movement of the indenter. Grain rearrangement and consolidation occur, leading to the formation of sintering necks. As temperature and pressure increase, ceramic grains develop stronger intergranular bonds, enhancing mass transport and accelerating densification within the matrix. The initial deceleration in indenter displacement during this stage is associated with the evaporation of trace amounts of B_2_O_3_, which is released as a gaseous byproduct. The third and final stage commences at 1800 °C and persists until the holding period. In the final stage, the indenter displacement stabilizes, exhibiting a gradual downward trend with minimal movement. This behavior reflects the establishment of an equilibrium between the thermomechanical effects (temperature and pressure) and diffusion-driven densification mechanisms (volume and grain boundary diffusion). Continuous densification occurs as temperature and pressure are maintained, facilitating further grain boundary migration and pore elimination.

**Figure 4:**
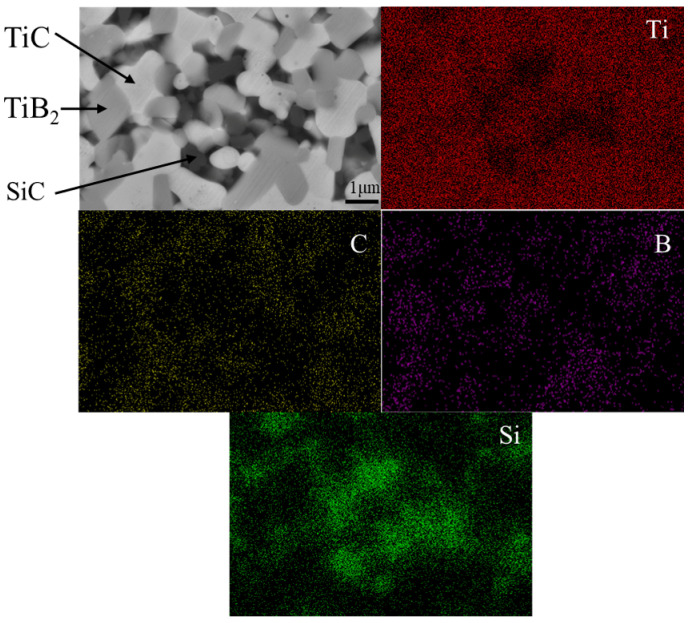
SEM image and element distribution of TiB_2_–TiC–SiC composite ceramics obtained at 1850 °C.

Figure 3b presents the X-ray diffraction (XRD) patterns of TiB_2_–TiC–SiC multiphase ceramics prepared at various sintering temperatures. All samples exhibit diffraction peaks corresponding to the TiB_2_, TiC, and SiC phases, indicating that variations in sintering temperature did not induce phase transformations. This observation is further corroborated by energy-dispersive spectroscopy (EDS) analysis. Figure 4 depicts elemental distribution maps obtained via EDS area scanning, revealing the presence of four primary elements: Ti, B, C, and Si. Notably, the spatial distributions of B, C, and Si exhibit complementary patterns. Specifically, dark gray regions correspond to B enrichment, suggesting the formation of the TiB_2_ phase, while light gray areas indicate C accumulation, implying the presence of the TiC phase. Additionally, black regions denote Si enrichment, consistent with the SiC phase. As the sintering temperature increased from 1800 °C to 1900 °C, the intensity of the TiB_2_ diffraction peaks progressively increased relative to those of TiC. This phenomenon is attributed to the reaction between TiC and impurities (e.g., B_2_O_3_ and residual carbon) present in the multiphase powder during sintering, which promotes the formation of additional TiB_2_. The reaction can be described as follows:

TiC + B_2_O_3_ + 2C = TiB_2_ + 3CO(g) (3)

**Figure 5:**
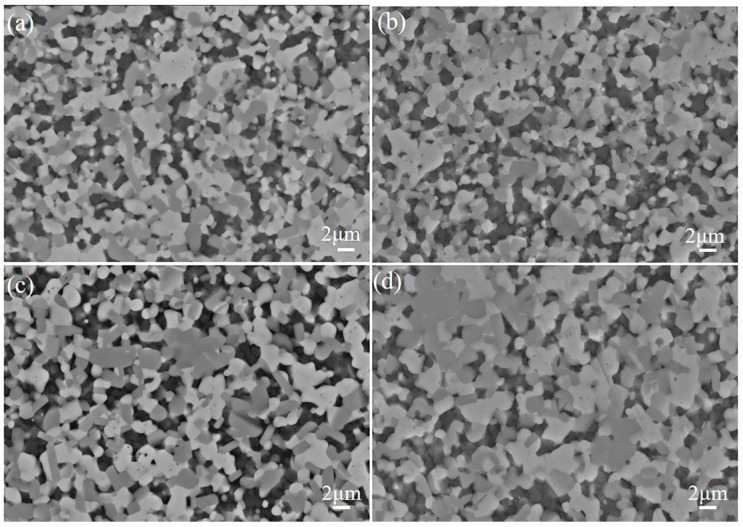
SEM images of TiB_2_–TiC–SiC composite ceramics obtained at different temperatures: (**a**) 1800 °C; (**b**) 1850 °C; (**c**) 1900 °C; (**d**) 1950 °C.

Figure 5 presents scanning electron microscopy (SEM) micrographs of the polished surfaces of TiB_2_–TiC–SiC composite ceramics sintered at varying temperatures. The images reveal distinct, tightly bonded grain boundaries between the TiC and TiB_2_ phases, forming a coherent composite matrix. In contrast, SiC particles aggregate at the grain boundaries of the TiB_2_–TiC phases, exhibiting weak interfacial bonding with TiC and TiB_2_. This poor adhesion is attributed to insufficient diffusion of silicon ions at grain boundaries and within grains during solid-state sintering, as no liquid-phase additives are employed. Consequently, grain boundary mobility is limited, hindering densification via solid-phase sintering mechanisms. Notably, the TiB_2_ grains in the as-received TiB_2_–TiC powder predominantly exhibit hexagonal or columnar morphologies. However, post-sintering, the TiB_2_ grains adopt a predominantly elongated rod-like structure. This morphological evolution significantly enhances the fracture toughness of the ceramic composite, as rod-like grains effectively impede crack propagation through mechanisms such as crack deflection and bridging.

A comparative analysis of microscopic images from samples sintered at varying temperatures reveals a temperature-dependent increase in grain size. Below 1900 °C, grain growth remains minimal; however, a substantial increase in grain size is observed when the sintering temperature exceeds 1900 °C. Elevated temperatures promote the progressive expansion of the contact area between TiC and TiB_2_ phases, fostering stronger intergranular bonding and facilitating densification. Notably, the distribution uniformity of SiC particles within the ceramic matrix deteriorates with increasing temperature. At 1900 °C, SiC particles exhibit the most homogeneous distribution, whereas higher temperatures lead to pronounced segregation. This phenomenon is attributed to the lattice mismatch between SiC particles and the TiB_2_–TiC matrix, which drives SiC particles toward grain boundary regions, resulting in localized enrichment and macroscopic nonuniformity in the matrix.

Table 3 summarizes the relative density and mechanical properties of TiB_2_–TiC–SiC multiphase ceramics sintered at varying temperatures. As shown in the table, the relative density initially increases with temperature but subsequently decreases beyond a critical threshold. Elevated temperatures enhance diffusion kinetics during solid-state sintering, promoting more uniform dispersion of SiC within the ceramic matrix. These factors facilitate densification, explaining the observed increase in relative density at lower sintering temperatures. However, when the sintering temperature exceeds 1900 °C, a decline in density occurs. This reduction, as illustrated in Figure 4, is primarily attributed to excessive grain growth, which induces pore accumulation at grain boundaries that cannot be effectively eliminated. Additionally, the deteriorating uniformity of SiC distribution within the matrix further exacerbates this density reduction.

Upon an analysis of the table, it becomes evident that the temperature-dependent trends in Vickers hardness, flexural strength, and fracture toughness of the composite ceramics closely correlate with variations in relative density. A positive correlation exists between the hardness of the ceramics and their internal compactness. Specifically, at a sintering temperature of 1900 °C, SiC exhibits relatively homogeneous dispersion within the ceramic matrix, minimizing pore formation and achieving the highest relative density. This optimized microstructure contributes to the maximum Vickers hardness observed at this temperature. Furthermore, the fracture toughness of the ceramics is influenced by the grain orientation relative to the applied load during fracture. The superior fracture toughness of the material sintered at 1900 °C suggests that this temperature effectively suppresses excessive grain growth, preserving a microstructure conducive to toughening mechanisms such as crack deflection and bridging.

### 3.2. The Impact of SiC Content on TiB_2_–TiC–SiC Ceramics

**Figure 6:**
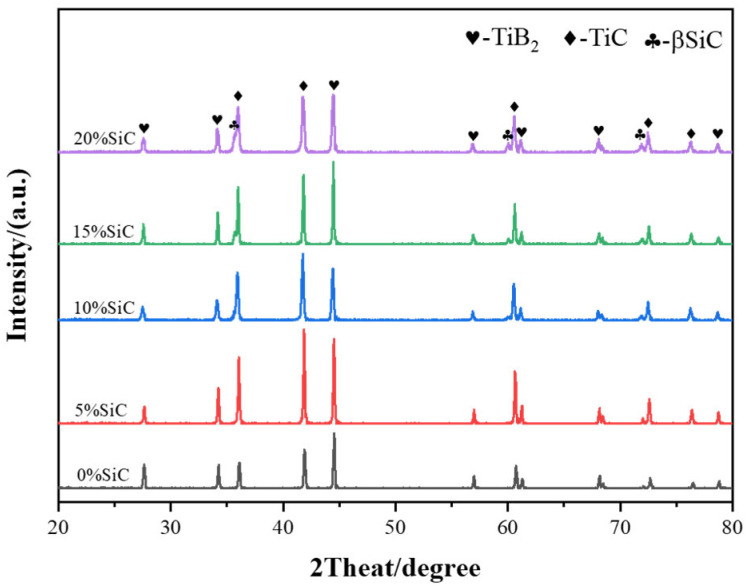
XRD diffraction patterns of composite TiB_2_–TiC–SiC ceramics fabricated with varying SiC concentrations.

**Figure 7:**
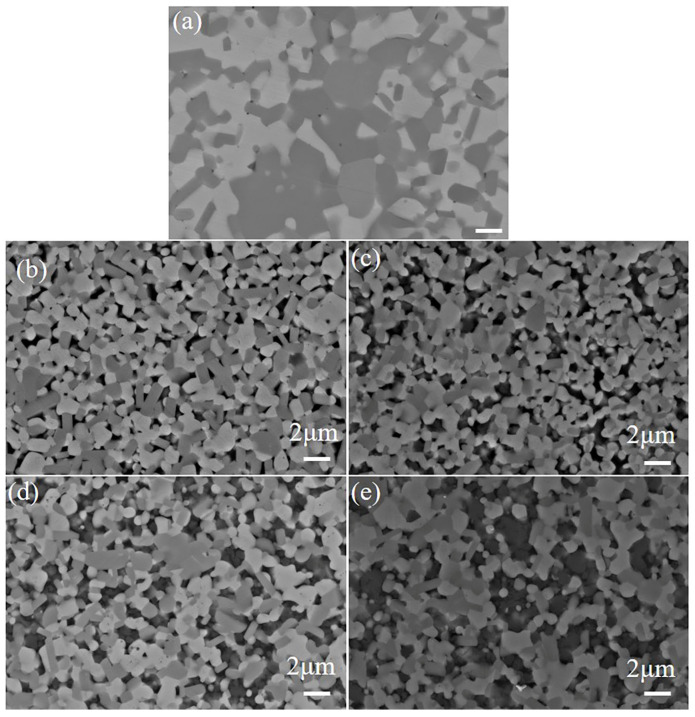
SEM images of composite ceramics with different SiC contents: (**a**) 0% SiC; (**b**) 5% SiC; (**c**) 10% SiC; (**d**) 15% SiC; (**e**) 20% SiC.

Figure 6 presents the X-ray diffraction (XRD) patterns of TiB_2_–TiC–SiC ceramics fabricated with varying SiC concentrations. As shown in the figure, increasing SiC content leads to a proportional increase in the intensity of the β–SiC diffraction peak, with no evidence of new phase formation. Figure 7 displays scanning electron microscopy (SEM) micrographs of the polished surfaces of TiB_2_–TiC–SiC ceramics. Compared to binary TiB_2_–TiC ceramics, the incorporation of SiC as a tertiary phase significantly refines the microstructure, reducing the average grain size from ~2 μm to ~1 μm. This observation highlights SiC’s efficacy in inhibiting grain growth. Additionally, SiC introduction increases the aspect ratio of TiB_2_ grains, which is beneficial for enhancing the fracture toughness of the multiphase ceramics. The formation of rod-like TiB_2_ grains can be attributed to two primary mechanisms. First, the unique sintering mechanism of spark plasma sintering (SPS) induces an electromigration effect, where the applied pulsed direct current drives directional diffusion of Ti and B ions along the electric field. Given the anisotropic hexagonal crystal structure of TiB_2_, ions exhibit higher diffusion rates along specific crystallographic planes, promoting preferential growth along the c-axis and resulting in a rod-like morphology. Second, the presence of SiC nanoparticles further enhances anisotropic grain growth. These nanoparticles, dispersed as a secondary phase within the TiB_2_ matrix, impede grain boundary migration via the Zener pinning effect. The pinning effect disproportionately restricts lateral (perpendicular to the pressure/electric field direction) grain boundary motion, thereby favoring longitudinal grain growth and exacerbating anisotropy. However, when the SiC content exceeds 15 wt%, significant SiC agglomeration occurs at TiB_2_–TiC grain boundaries, leading to nonuniform distribution and increased porosity at these interfaces.

Table 4 summarizes the relative density and mechanical properties of TiB_2_–TiC–SiC ceramics with varying SiC additions. As shown in the table, the fracture toughness of ternary TiB_2_–TiC–SiC composite ceramics is significantly enhanced compared to binary TiB_2_–TiC ceramics without SiC, confirming that SiC addition effectively improves ceramic toughness. Specifically, incorporating 15 wt% silicon carbide (SiC) increases the fracture toughness of the multiphase ceramics from 4.16 ± 0.24 MPa·m^1/2^ to 5.71 ± 0.34 MPa·m^1/2^. This enhancement is attributed to the Zener pinning effect induced by SiC, which refines the microstructure and promotes the formation of rod-shaped TiB_2_ grains. However, the flexural strength of the ceramics decreases due to weak interfacial bonding between SiC and the TiC–TiB_2_ matrix. Notably, the relative density, flexural strength, Vickers hardness, and fracture toughness of the material exhibit a non-monotonic trend, initially increasing and then decreasing with increasing SiC content, with optimal performance observed at 15 wt% SiC. A microstructural analysis suggests that an appropriate SiC content facilitates ceramic densification by refining grains and promoting uniform distribution. However, excessive SiC addition leads to agglomeration, resulting in poor interfacial bonding between agglomerates and surrounding grains. This generates a high density of intergranular pores, increasing porosity and degrading both the relative density and mechanical properties of the ceramics.

**Figure 8:**
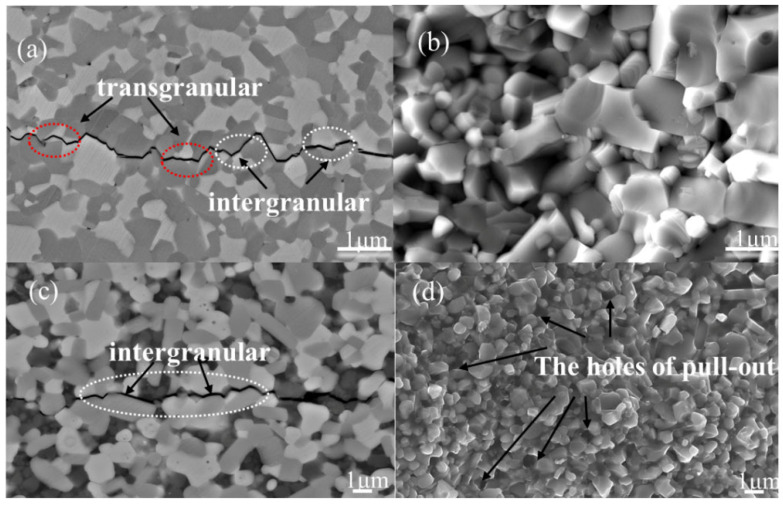
SEM images of TiB_2_–TiC binary and TiB_2_–TiC–SiC ternary multiphase ceramics. (**a**) Crack propagation in TiB_2_–TiC multiphase ceramics. (**b**) Bending fracture surface of TiB_2_–TiC multiphase ceramics. (**c**) Crack propagation in TiB_2_–TiC–SiC ternary multiphase ceramics. (**d**) Bending fracture surface of TiB_2_–TiC–SiC ternary multiphase ceramics.

Figure 8 presents scanning electron microscopy (SEM) micrographs depicting crack propagation and bending fracture surfaces in (a) TiB_2_–TiC binary ceramics (without SiC) and (c) TiB_2_–TiC–SiC ternary ceramics (with 15 wt% SiC). In the binary system (Figure 8a), crack propagation exhibits a mixed-mode fracture behavior, combining intergranular and transgranular fracture paths. Specifically, intergranular fractures dominate at the phase boundaries between TiC and TiB_2_, while transgranular fractures are more prevalent within the TiC phase. This behavior is attributed to the lower intrinsic fracture toughness of the TiC phase compared to TiB_2_. In contrast, the ternary system (Figure 8c) predominantly displays intergranular fractures, distinct from the binary counterpart. This shift in fracture mode arises from the relatively weak interfacial bonding between SiC and the TiB_2_–TiC matrix. As a result, any applied stress is primarily dissipated at the grain boundaries, promoting crack propagation along the interfacial regions of the TiB_2_, TiC, and SiC phases. This stress-relief mechanism effectively dissipates the energy associated with applied loads, thereby enhancing the ceramic’s fracture toughness. Additionally, Figure 8d highlights significant grain pull-out, particularly of SiC grains, further corroborating the weak interfacial bonding between SiC and the TiB_2_/TiC matrix.

**Figure 9:**
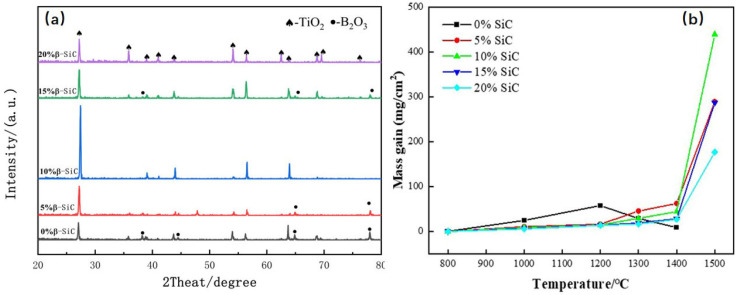
Composite ceramics with different SiC contents: (**a**) XRD patterns of the oxide layer; (**b**) oxidation weight gain curves.

Single-phase SiC exhibits exceptional oxidation resistance, primarily due to the formation of an amorphous SiO_2_ passivation layer during oxidation. This SiO_2_ layer acts as a protective barrier, enabling SiC ceramics to withstand oxidation at temperatures up to 1600 °C in ambient air. Consequently, the incorporation of SiC is expected to enhance the high-temperature oxidation resistance of TiB_2_–TiC ceramics. To evaluate this improvement, static oxidation tests were conducted to measure the oxidation resistance of (1) TiB_2_–TiC binary ceramics (without SiC) and (2) TiB_2_–TiC–SiC ternary ceramics (with 15 wt% SiC). Figure 9 presents the X-ray diffraction (XRD) patterns of the oxide layers formed on these ceramics after oxidation at 1200 °C.

The hypothesized oxidation reactions for the TiC phase within TiB_2_–TiC–SiC composite ceramics at elevated temperatures are

TiC + O_2_(g) = TiO_2_ + C (4)

TiC + 2O_2_(g) = TiO_2_ + CO_2_(g) (5)

Similarly, the postulated oxidation reactions for the TiB_2_ phase at high temperatures are

TiB_2_ + 2O_2_(g) = TiO + B_2_O_3_ (6)

2TiB_2_ + 5O_2_(g) = 2TiO_2_ + 2B_2_O_3_ (7)

4TiB_2_ + 9O_2_(g) = 2Ti_2_O_3_ + 4B_2_O_3_ (8)

2Ti_2_O_3_ + O_2_(g) = 4TiO_2_ (9)

2TiO + O_2_(g) = 2TiO_2_ (10)

Depending upon the oxygen concentration, active oxidation of silicon carbide occurs at oxygen pressures less than one bar according to the following equation [26]:

SiC + O_2_(g) = SiO_2_(g) + CO_2_(g) (11)

**Figure 10:**
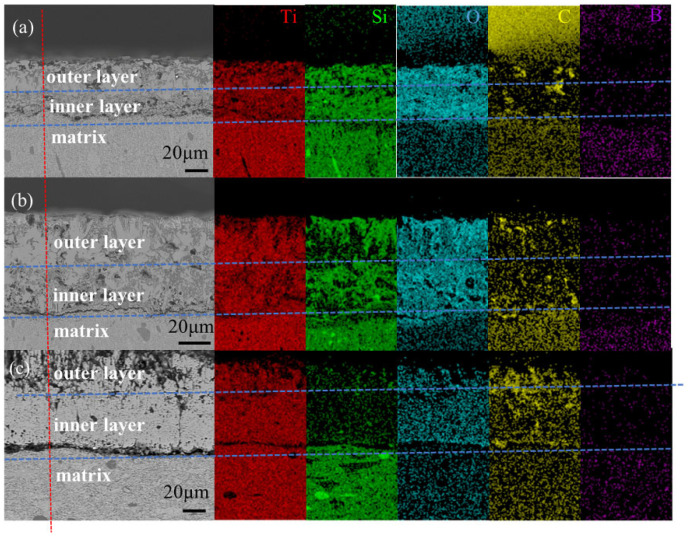
SEM and EDS images of TiB_2_–TiC–SiC composite ceramics with 15% SiC addition after oxidation at different temperatures: (**a**) 1200 °C, (**b**) 1300 °C, (**c**) 1400 °C.

Based on the reaction equations, the anticipated oxidation products of ternary TiB_2_–TiC–SiC ceramics include TiO_2_, SiO_2_, and B_2_O_3_. However, as shown in the XRD patterns, the oxide layer predominantly consists of TiO_2_, with only trace amounts of B_2_O_3_ detected. This discrepancy can be attributed to the crystal-to-amorphous phase transition of SiO_2_ and B_2_O_3_ during rapid cooling, rendering these phases undetectable by XRD diffraction on the sample surface. Additionally, the high-temperature oxidation experiments were conducted under oxygen-rich conditions, promoting active oxidation of silicon carbide, as described by reaction (11). The SiO_2_ formed undergoes vaporization upon formation, contributing to mass loss. Energy-dispersive X-ray spectroscopy (EDS) mapping of the sample oxidized at 1400 °C (Figure 10) reveals a significant depletion of silicon (Si) in the outer oxidation layer, consistent with this hypothesis.

Figure 9b illustrates the oxidation weight gain curves for composite ceramics. As shown, the SiC-free ceramics exhibit significant oxidation weight gain starting at 800 °C, followed by weight loss above 1200 °C due to the accelerated vaporization of liquid B_2_O_3_, which disrupts the protective oxide layer formed by B_2_O_3_. In contrast, SiC-containing ceramics demonstrate a gradual weight gain up to 1400 °C, indicating that the SiO_2_ layer derived from SiC oxidation effectively protects the matrix and suppresses B_2_O_3_ evaporation. However, above 1400 °C, enhanced B_2_O_3_ evaporation and damage from gaseous oxidation byproducts compromise the oxide layer, leading to complete oxidation by 1500 °C. Notably, SiC-free ceramics remain stable only up to 1200 °C in oxygen-rich environments, whereas SiC-reinforced ternary composites retain stability up to 1400 °C. This suggests that SiC addition improves oxidation resistance by 200 °C. Among ceramics with varying SiC contents, the 20 wt% SiC sample exhibits superior oxidation resistance, attributed to the formation of a thicker SiO_2_ passivation layer that enhances matrix protection.

To investigate the oxidation behavior of composite ceramics, Figure 10 presents cross-sectional scanning electron microscopy (SEM) images and energy-dispersive X-ray spectroscopy (EDS) elemental maps of a 15 wt% SiC-containing ceramic oxidized at temperatures ranging from 1200 °C to 1400 °C for 8 h. It is noteworthy that the surface oxide layer of the sample oxidized at 1500 °C delaminated, precluding further analysis. Observations indicate that the oxide layer formed during oxidation is nonuniform, with visible pores attributed to gas evolution during the process. The EDS analysis reveals that the oxide layer primarily consists of Ti, Si, O, C, and B. Elemental mapping of oxygen (O) confirms the presence of two distinct sublayers: an outer oxide layer and an inner oxide layer. The outer oxide layer predominantly contains Ti, Si, B, and O, corresponding to oxidation products B_2_O_3_, TiO_2_, and SiO_2_. The low B content in the outer layer is attributed to B_2_O_3_ vaporization. In contrast, the inner oxide layer exhibits a more porous and loosely packed structure, with numerous cavities. As the oxidation temperature increases, the thickness of the inner oxide layer grows, and cavities coalesce to form interconnected channels, indicative of extensive oxidation.

The EDS analysis reveals the presence of carbon (C) in the oxidation layer, in addition to the oxidation products TiO_2_, SiO_2_, and B_2_O_3_. This observation is consistent with the oxidation of SiC and TiC, as described by reactions (2) and (9). The primary oxidation resistance mechanisms in TiB_2_–TiC–SiC ternary multiphase ceramics involve two critical factors. First, the formation of an amorphous SiO_2_ passivation layer derived from oxidation products effectively impedes oxygen ingress into the matrix. Second, the co–melting of B_2_O_3_ and SiO_2_ generates borosilicate glass, increasing the surface glass phase content. As a result, a dense oxidation film, comprising a glassy phase and crystalline TiO_2_, forms on the sample surface, providing robust protection to the interior and mitigating rapid oxidation.

## 4. Conclusions

Guided by the practical demands of high-temperature applications, including crucibles for metallurgical processes, aerospace thermal protection systems, and nuclear reactor cladding materials, this study aims to develop high-temperature TiB_2_–TiC–SiC structural ceramics with superior oxidation resistance and balanced mechanical properties. To this end, the effects of sintering temperature and SiC addition on the microstructure, mechanical performance, and oxidation resistance of TiB_2_–TiC–SiC composite ceramics were systematically investigated. The key findings from this study are summarized as follows:

(1) The densification of TiB_2_–TiC–SiC composite ceramics follows a typical solid-phase sintering mechanism, progressing through three distinct stages. An optimal sintering temperature of 1900 °C was identified, at which the material exhibits uniform phase distribution, controlled grain growth, and strong intergranular bonding. This temperature yields maximum densification and mechanical performance.

(2) The incorporation of an appropriate amount of SiC enhances the microstructure, mechanical properties, and oxidation resistance of the composite ceramics. Compared to binary TiB_2_–TiC ceramics, the ternary composites exhibit improved fracture toughness, attributed to SiC-mediated grain refinement and the formation of rod-like TiB_2_ grains. Notably, a higher SiC content correlates with enhanced oxidation resistance, likely due to the formation of protective oxide layers during high-temperature exposure.

## Figures and Tables

**Table 1:** Temperature optimization scheme for TiB_2_–TiC–SiC ternary multiphase ceramics.

Sample	Sintering Temperature (°C)	Time (min)	Pressure (MPa)
15% SiC–1800	1800	10 min	40 Mpa
15% SiC–1850	1850	10 min	40 Mpa
15% SiC–1900	1900	10 min	40 Mpa
15% SiC–1950	1950	10 min	40 Mpa

**Table 2:** Formulation scheme for TiB_2_–TiC–SiC ternary multiphase ceramics.

Sample	TiB_2_	TiC	SiC
0% SiC	44.25%	55.5%	0%
5% SiC	42.275%	52.725%	5%
10% SiC	40.05%	49.95%	10%
15% SiC	37.825%	47.175%	15%
20% SiC	35.6%	44.4%	20%

**Table 3:** Mechanical properties of TiB_2_–TiC–SiC composite ceramics sintered at different temperatures.

Temperature (°C)	Relative Density (%)	Flexural Strength (MPa)	Vickers Hardness (GPa)	Fracture Toughness (MPa·m^1/2^)
1800	96.54%	359.51 ± 14.7	20.91 ± 0.51	4.98 ± 0.24
1850	97.08%	374.16 ± 41.2	21.59 ± 0.73	5.01 ± 0.12
1900	98.76%	435.98 ± 25.3	22.20 ± 0.43	5.71 ± 0.34
1950	98.08%	432.64 ± 34.7	21.91 ± 0.62	5.06 ± 0.24

**Table 4:** Mechanical properties of composite ceramics with different SiC contents.

Temperature (°C)	Relative Density (%)	Flexural Strength (MPa)	Vickers Hardness (GPa)	Fracture Toughness (MPa·m^1/2^)
0% SiC	98.56%	509.44 ± 31.5	22.10 ± 0.36	4.16 ± 0.24
5% SiC	97.49%	247.76 ± 17.3	20.99 ± 0.45	4.20 ± 0.25
10% SiC	97.99%	410.90 ± 31.5	22.05 ± 0.63	4.55 ± 0.36
15% SiC	98.76%	435.98 ± 25.3	22.20 ± 0.43	5.71 ± 0.34
20% SiC	98.10%	417.14 ± 22.3	22.03 ± 0.47	5.42 ± 0.41

## Data Availability

The original contributions presented in this study are included in the article. Further inquiries can be directed to the corresponding author(s).
